# The diagnostic value of rapid urine test platform UF-5000 for suspected urinary tract infection at the emergency department

**DOI:** 10.3389/fcimb.2022.936854

**Published:** 2022-09-27

**Authors:** Tsun Tsun Stacia Chun, Xiaohao Ruan, Sau Loi Ng, Hoi Lung Wong, Brian Sze Ho Ho, Chiu Fung Tsang, Terence Chun Ting Lai, Ada Tsui Lin Ng, Wai Kit Ma, Wayne Pei Lam, Rong Na, James Hok Leung Tsu

**Affiliations:** ^1^ Division of Urology, Department of Surgery, The University of Hong Kong, Hong Kong, Hong Kong SAR, China; ^2^ Department of Urology, Ruijin Hospital, Shanghai Jiaotong University School of Medicine, Shanghai, China; ^3^ Division of Urology, Department of Surgery, Queen Mary Hospital, Hong Kong, Hong Kong SAR, China; ^4^ Hong Kong Urology Clinic, Hong Kong, Hong Kong SAR, China

**Keywords:** bacteria, dipstick, Sysmex UF-5000, urine culture, urinary tract infection

## Abstract

**Background and objective:**

Urine culture is time consuming, which may take days to get the results and impede further timely treatment. Our objective is to evaluate whether the fast urinalysis and bacterial discrimination system called Sysmex UF-5000 may predict urinary tract infections (UTIs) (within minutes) compared with the clinical routine test in suspected UTI patients. In addition, we aimed to explore the accuracy of microbiologic information by UF-5000.

**Materials and Methods:**

Consecutive patients who were admitted from the emergency department at Queen Mary Hospital (a tertiary hospital in Hong Kong) from June 2019 to February 2020 were enrolled in the present study. The dipstick test, manual microscopic test with culture, and Sysmex UF-5000 test were performed in the urine samples at admission.

**Results:**

A total of 383 patients were finally included in the present study. UF-5000 urinalysis (area under the receiver operator characteristic curve, AUC=0.821, confidence interval, 95%CI: 0.767–0.874) outperformed the dipstick test (AUC=0.602, 95%CI: 0.550–0.654, P=1.32×10^-10^) for predicting UTIs in patients without prior antibiotic treatment. A significant net benefit from UF-5000 was observed compared with the dipstick test (NRI=39.9%, 95%CI: 19.4–60.4, P=1.36 × 10^-4^). The urine leukocyte tested by UF-5000 had similar performance (AUC) for predicting UTI compared with the manual microscopic test (P=0.27). In patients without a prior use of antibiotics, the concordance rates between UF-5000 and culture for predicting Gram-positive or -negative bacteriuria and a negative culture were 44.7% and 96.2%, respectively.

**Conclusions:**

UF-5000 urinalysis had a significantly better predictive value than the dipstick urine test for predicting UTIs.

## Background

Urinary tract infection (UTI) is one of the most common diseases in the population (Foxman, 2002; Gupta et al., 2017). It accounts for an incidence of 1.75% among the population over 18 years old in North America (Laupland et al., 2007). In the United States, it is estimated that 143 million patients visit hospitals for UTIs every year since 2018, making UTI the seventh most common reason for emergency visits (Zilberberg et al., 2022a). A complicated UTI is also one of the most common reasons of hospitalization from the emergency department, which brings a significant socioeconomic burden to the society (Zilberberg et al., 2022b).

Urine dipstick and machine-based urinalysis are the two most common ways of urinary test for clinical practice (Oyaert and Delanghe, 2019; Kavuru et al., 2020). Samples [i.e., mid-stream urine (MSU)] are usually sent to a urinary culture thereafter for the identification of bacteria and antibiotic susceptibility test. The result of urinalysis can usually be obtained within minutes or hours. It provides some important information for clinical practice, for example, white blood cells (WBCs) and red blood cells (RBCs). However, other essential information for clinical intervention such as the bacteria strain and drug sensitivity cannot be obtained until days later. This may lead to a delayed treatment with effective antibiotics.

In recent years, automated flow cytometry–based urine sediment analyzers have provided a faster and more accurate way to detect bacteriuria (Moshaver et al., 2016). It is cost-effective, and the results may be reported within minutes (Moshaver et al., 2016). To further provide evidence for antibiotic treatment, a urine sediment analyzer called UF-5000 (Sysmex Corporation, Kobe, Japan) can also perform bacterial discrimination for Gram-positive (G+) or Gram-negative (G-) flags with higher accuracy compared with a quick Gram stain (Enko et al., 2021). This instrument may not only help microbiologists better identify patients with suspected UTIs but also provide initial/fast (within minutes) evidence for antibiotic treatment (Ippoliti et al., 2020).

To better understand the utility of UF-5000 in suspected UTIs, we conducted the current observational case–control study in a single tertiary medical center in Hong Kong, China. In clinical practice, hospitalized suspected UTIs in Hong Kong are complicated UTIs and always come with certain symptoms, such as fever (≥37.3°C) and loin pain. The performance of UF-5000 in diagnosis and bacterial discrimination among suspected UTIs at the emergency department will be evaluated compared with the dipstick quick test and urine culture of MSU.

## Methods

### Study population

Consecutive patients who were admitted from the emergency department at Queen Mary Hospital (a tertiary hospital in Hong Kong) from June 2019 to February 2020 were enrolled in the present study. The inclusion criterion was patients suspected to have UTIs who were referred by a primary care physician or might present symptoms including dysuria, urinary frequency, gross or microscopic hematuria, foul-smelling urine, fever, and lower abdominal/loin pain. Written consent from each of the patients was obtained. The study was approved by the institutional review board of the University of Hong Kong and Hospital Authority Hong Kong West Cluster (UW 19-037).

### Urinalysis, urine culture, and UF-5000

A urine sample (~10 ml) was collected from each of the patients in the form of mid-stream, catheter, and percutaneous nephrostomy in sterile Vacuette Urine CCM tubes. Urine specimens were not suitable for further analysis if they (1) have a high concentration of mucus strands; (2) have fluorescent matter due to the inclusion of chemicals; (3) contain preservatives; and (4) cannot be sent immediately to the lab or stored in 4°C refrigerators over 12 h. Samples were then sent for the dipstick test, laboratory routine microscopy (manual), urine culture, and Sysmex UF-5000 analysis.

The dipstick test was performed using URISCAN PRO II system (YD Electronics, Yongin-si, Korea) (Kavuru et al., 2020). A manual microscopic test (for white blood cells) was performed by the microbiologists on duty. Quantitative urine culture was performed with a standard protocol by Dr. Jonathan Chen at the Department of Microbiology of the hospital. A standard protocol was performed inoculating 10 μl of a well-mixed urine specimen by a sterile disposable polystyrene loop onto ChromID CPS Elite (BioMerieux, Marcy l’Etoile, France), 5% horse blood agar, MacConkey agar, or Cystine-lactose-electrolyte-deficient CLED, agar. The plates were incubated aerobically at 35°C overnight, and growth was examined on both plates; if no growth is observed, the plates are incubated for another 24 h. The results were expressed as the number of colony-forming units per milliliter (CFU/ml). The amount of bacterial growth was assessed as no growth, <10^4^ CFU/ml, 10^4^–10^5^ CFU/ml, and >10^5^ CFU/ml (according to Hong Kong laboratory standards). The isolated microorganisms were then assessed by professional microbiology doctors and classified into three kinds: concrete species (including Gram-positive, Gram-negative, and non-bacteria), mixed flora (more than one isolate or mixed gram pattern), and undetectable.

Sysmex UF-5000 is a fluorescence flow cytometric–based analyzer that may provide routine microscopy analysis and bacteria information within 3 h with different categories including the bacteria count, G+ (marked as “Gram Positive?” by the system), G- (“Gram Negative?”), G+/- (both, “Gram Pos/Neg?”), and “unclassified” (indicating no bacteria or unable to detect, “unclassified,” or the absence of bacterial information). The breakpoint for bacteria was 196 bacteria/μl, and, for WBC, it was 14.7 cells/μl in the Sysmex analysis. The rinsing steps between samples were used in all analyses. If the high values of the three parameters were detected (BACT ≥ 1,000/μl; WBC/RBC ≥ 10,000/μl), the anticarryover function (auto additional rinse) will operate. This series was consecutively analyzed three times, followed by a triplicate of specimens with very low values (blank). The carryover rate was determined by the formula carryover = (blank 1–blank 3)/(sample 3–blank 3) for all three runs, and mean values were calculated for each parameter. Then, the specification for carryover in BACT is 0.05% or less; if the BACT count = 10,000/μl, the maximum carryover will be 5.0/μl. The measuring interval and display range of BACT in UF-5000 is 5.0–10,000/μl and 0–99,999/μl, respectively.

UTI is an infection in any part of the urinary system (kidneys, ureters, bladder, and urethra). The gold standard for diagnosing UTIs is urine culture and the microbiological confirmation of etiologic bacteria. In the current study, UTI (significant bacteriuria, in other words) was defined stringently as the growth of a single and same pathogenic organism at a concentration of ≥10^5^ CFU/ml (Lough et al., 2019). Suspected contamination was defined as mixed flora at a concentration of ≥10^4^ CFU/ml (Wilson and Gaido, 2004; Lough et al., 2019; Ippoliti et al., 2020; Guri et al., 2021).

### Statistical analysis

Baseline characteristics were illustrated by descriptive statistics. A chi-square test was used to compare the differences of categorical variables. Model discrimination was assessed with the area under the receiver operating characteristic curve (AUC). The Youden index was used to determine the optimal cut-point of the BACT/WBC parameters. AUCs were compared between two correlated receiver operating characteristic (ROC) curves by the theory on generalized U-statistics (Delong et al., 1988). Model performance was also assessed with net reclassification improvement (NRI) (Jewell et al., 2016). A Z-test was used to test for the null hypothesis of NRI = 0. The concordance rate was applied to compare the rate of agreement in Gram staining between UF-5000 and the culture of MSU. All statistical analyses were performed using R version 4.1.2, (R Core Team, 2021) and a two-tailed p < 0.05 was considered statistically significant.

## Results

A total of 383 patients were finally included in the present study. Among them, 90 (23.5%) had significant bacteriuria from a urine culture and were classified into the laboratory-supported UTI group. In the UTI group, the majority of the microorganisms isolated were *Escherichia coli* (51.1%) followed by *Enterococcus faecalis* (12.2%). *Staphylococcus* species, *Streptococcus* species, and *Klebsiella pneumonia* were the third most common species (6.7%, **Supplementary Table 1**). Baseline characteristics and urine test results are presented in **Table 1**. In total, 28.5% of the patients were women and 68.9% were men. UTI-related symptoms were observed in 250 (65.3%) patients including dysuria, loin pain, and fever. A total of 104 (27.2%) were prescribed with antibiotics within 2 weeks before admission. In total, 68.4%, 31.1%, and 0.5% of the patients’ urine specimens were mid-stream, catheter urine, and percutaneous nephrectomy, respectively. In addition, patients who were women and presented with fever or dysuria were more likely to get positive urine culture results (P<0.05, **Table 1**). Finally, 282, 372, and 383 urine samples were able to be analyzed by laboratory microscopy, a dipstick test, and the UF-5000 system, respectively.

**Table 1:** Baseline clinical characteristics of patients with or without urinary tract infection (urine culture support).

Patient characteristics	Patients with UTI^a^	Patients without UTI	All	P-value
**No. of participants**	90	293	383	–
**Sex, No. (%)**				2.79×10^-5^
Men	45 (50.0)	219 (74.7)	264 (68.9)	
Women	41 (45.6)	68 (23.2)	109 (28.5)	
Missing	4 (4.4)	6 (2.1)	10 (2.6)	
**Age, mean (SD)**	65.3 (18.5)	63.4 (17.6)	63.8 (17.8)	0.39
**Symptoms, No. (%)**	61 (67.8)	189 (64.5)	250 (65.3)	
Dysuria, n	24 (26.7)	37 (12.6)	61 (15.9)	1.77×10^-3^
Loin pain, n	22 (24.4)	84 (28.7)	106 (27.7)	0.25
Fever, n	17 (18.9)	24 (8.2)	41 (10.7)	5.40×10^-3^
Missing	29 (32.2)	104 (35.5)	133 (34.7)	–
**Prior antibiotics,** n^b^, No. (%)	19 (21.1)	85 (29.0)	104 (27.2)	0.17
**Urine specimen type, No. (%)**				0.08
Mid-stream	55 (61.1)	207 (70.6)	262 (68.4)	
Catheter	34 (37.8)	85 (29.0)	119 (31.1)	
Percutaneous nephrectomy	1 (1.1)	1 (0.4)	2 (0.5)	

UTI, urinary tract infection; IQR, interquartile range; WBC, white blood cell in urine; RBC, red blood cell in urine.

a UTI was diagnosed as the growth of a single and same pathogenic organism at aconcentration of >105 colony-forming units per milliliter (CFU/ml) in urine samples.

b Within 2 weeks prior to inclusion.

We then evaluated the predictive value of UF-5000 for UTIs compared with a dipstick. UF-5000 urinalysis had higher sensitivity but relatively lower specificity compared with the dipstick (**Tables 2**, **4**). For example, both nitrite (NIT, indicates bacteriuria) and white blood cells (WBCs) positive from the dipstick test had 24.1% sensitivity and 96.5% specificity for predicting UTIs, while bacteriuria and WBC detected by UF-5000 would have 79.8% sensitivity and 76.9% specificity (the cutoff points of the WBC and BACT parameters were determined by the Youden index: WBC=14.7 cells/μl, BACT=196 unit/μl; **Table 2**). This indicated that the dipstick would have a relatively higher false-negative rate, which might lead to delayed treatment (**Table 3**). Bacteriuria and WBCs detected by UF-5000 had an AUC of 0.783 (95% confidence interval, 95%CI: 0.734–0.833), which was significantly higher than those detected by the dipstick test (AUC=0.603, 95%CI: 0.557–0.650, P=1.61×10^-9^). Net reclassification improvement analysis (NRI) showed a significant net benefit from using UF-5000 analysis for predicting UTIs compared to the dipstick test (bacteriuria and WBCs by UF-5000), causing 36.0% of individuals (NRI, 95%CI: 18.8–53.2, P= 3.94×10^-5^, **Table 2**) to be reclassified into the correct decision (both negative and positive UTIs). Subgroup analysis was also performed in patients without prior antibiotic treatment (within 2 weeks before admission; **Table 4**). UF-5000 urinalysis performed even better among these patients for predicting UTIs with higher sensitivity (84.1%) and specificity (80.0%). In addition, bacteriuria and WBC detected by UF-5000 had a better AUC of 0.821 (95%CI: 0.767–0.874) than those detected by the dipstick test (AUC=0.602, 95%CI: 0.550–0.654, P=1.32×10^-10^; **Table 4**). A significant net benefit from UF-5000 was also observed compared with the dipstick test (bacteriuria and WBCs by UF-5000, NRI=39.9%, 95%CI: 19.4–60.4, P=1.36 × 10^-4^; **Table 4**). Moreover, WBC identified by UF-5000 had similar performance (AUC) for predicting UTIs compared with the MSU manual microscopic test in all patients or patients without prior antibiotic treatment (P=0.47 and P=0.27, respectively).

**Table 3:** Biochemical indexes and bacterial information measured by three different tests.

Urine test characteristics	Patients with UTI^a^	Patients without UTI	All	P-value
**Biochemical indexes in urine**				
**MSU microscopy**	61	221	282	–
Pyuria, n^b^ (%)	24 (39.3)	11 (5.0)	35 (12.4)	2.10×10^-12^
Bacteriuria+, n^c^ (%)	90 (100.0)	11 (5.0)	101 (35.8)	9.72×10^-33^
Hematuria, n^d^ (%)	16 (26.2)	34 (15.4)	50 (17.7)	4.78×10^-5^
**Dipstick test**	87	285	372	–
WBC, urine (≥1+), n (%)	61 (70.1)	67 (23.5)	128 (34.4)	9.60×10^-16^
Nitrite (+), n (%)	24 (27.6)	19 (6.7)	43 (11.6)	8.49×10^-8^
RBC, urine (≥1+), n (%)	60 (76.0)	187 (69.5)	247(71.0)	0.25
**Sysmex UF-5000 test**	90	293	383	–
WBC (+)^e^, n (%)	85 (94.4)	148 (50.5)	233 (60.8)	8.15×10^-14^
RBC (+)^f^, n (%)	60 (66.7)	190 (64.9)	250 (65.3)	0.75
Bacteria count (+)^g^, n (%)	69 (76.7)	76 (25.9)	145 (37.9)	4.02×10^-18^
UTI detection, n (%)	86 (95.6)	165 (56.3)	251 (65.5)	7.32×10^-12^
**Gram’s staining result**				
**MSU culture**	90	293	383	–
Gram (+), n (%)	24 (26.7)	0	24 (6.3)	–
Gram (-), n (%)	65 (72.2)	2 (0.7)	67 (17.5)	–
Mixed or non-bacteria, n (%)	1 (1.1)	9 (3.1)	10 (2.6)	–
Undetectable, n (%)	0	282 (96.3)	282 (73.6)	–
**Sysmex UF-5000 test**	90	293	383	
Gram (+), n (%)	24 (26.7)	59 (20.1)	83 (21.7)	–
Gram (-), n (%)	29 (32.2)	17 (5.8)	46 (12.0)	–
Mixed, n (%)	28 (31.1)	41 (14.0)	69 (18.0)	–
Undetectable, n (%)	9 (10.0)	176 (60.1)	185 (48.3)	–
**Contamination**, n (%)	0	8 (2.7)	8 (1.8)	–

UTI, urinary tract infection; MSU, midstream urine; WBC, white blood cell; RBC, red blood cell.

a UTI was diagnosed as growth of a single and same pathogenic organism at a concentration of >10^5^ colony-forming units per milliliter (CFU/ml) in urine samples.

b Pyuria was defined as a WBC count of >10/μl.

c Any bacterium in the urine was considered as positive bacteriuria.

d Hematuresis was defined as an RBC count of >200/μl.

e WBC parameter (+) was set as WBC≥14.7 cells/μl.

f RBC parameter (+) was set as RBC≥20 cells/μl.

g BACT parameter (+) was set as BACT ≥196 unit/μl.

**Table 2:** Comparison of predictive ability of mid-stream urine (MSU) microscopy, the dipstick test, and the UF-5000 test in all patients (n=383).

Test	Variables	Sensitivity	Specificity	PPV	NPV	AUC (95%CI)	P-value	NRI,% (95%CI)	P-value
MSU	WBC	68.6%	85.0%	42.6%	95.0%	0.672 (0.608-0.735)	–	–	–
Dipstick	NIT	38.1%	93.4%	55.8%	80.9%	0.605 (0.555-0.654)	Ref^a^.	0	Ref^a^.
	WBC	70.1%	76.6%	47.7%	89.4%	0.733 (0.679-0.788)	Ref^b^.	0	Ref^b^.
	NIT and WBC	24.1%	96.5%	67.7%	80.7%	0.603 (0.557-0.650)	Ref^c^.	0	Ref^c^.
	NIT or WBC	73.6%	73.0%	45.4%	90.0%	0.733 (0.679-0.786)	Ref^d^.	0	Ref^d^.
UF-5000	BACT parameter^e^	82.1%	73.8%	47.6%	93.4%	0.781 (0.733-0.830)	1.91×10^-7^	34.9 (31.1-66.8)	1.23×10^-4^
	WBC parameter^f^	94.4%	49.5%	36.5%	96.7%	0.720 (0.682-0.757)	0.69	-2.9 (-15.3~9.6)	1.00
	BACT and WBC	79.8%	76.9%	50.0%	92.9%	0.783 (0.734-0.833)	1.61×10^-9^	36.0 (18.8-53.2)	3.94×10^-5^
	BACT or WBC	96.7%	47.1%	36.0%	97.9%	0.719 (0.685-0.753)	0.59	-3.6 (-16.1~8.8)	0.72

PPV, positive predictive value; NPV, negative predictive value; AUC, area under the receiver operating curve; NRI, net reclassification index; CI, confidence interval; NIT, nitrite; BACT, bacteria count; WBC, white blood cells in urine; Ref., Reference.

a Compare NIT and BACT;

b Compare dipstick WBC and UF-5000 WBC.

c Compare NIT and WBC and BACT and WBC.

d Compare NIT/WBC and BACT/BACT.

e Parameter (+) was set as BACT ≥196 units/μl.

f WBC parameter (+) was set as WBC ≥ 14.7 cells/μl.

**Table 4:** Comparison of predictive ability of MSU microscopy, the dipstick test, and the UF-5000 test in patients without taking prior antibiotics in 2 weeks (n=269).

Test	Variables	Sensitivity	Specificity	PPV	NPV	AUC (95%CI)	P-value	NRI,% (95%CI)	P-value
MSU	WBC	26.7%	97.3%	75.0%	81.3%	0.620 (0.553-0.686)	–	–	–
Dipstick	NIT	26.6%	96.4%	70.8%	80.2%	0.615 (0.559-0.671)	Ref^a^.	0	Ref^a^.
	WBC	67.2%	81.2%	53.8%	88.4%	0.742 (0.678-0.806)	Ref^b^.	0	Ref^b^.
	NIT and WBC	21.9%	98.5%	82.4%	79.5%	0.602 (0.550-0.654)	Ref^c^.	0	Ref^c^.
	NIT or WBC	71.9%	78.6%	52.3%	89.5%	0.752 (0.690-0.815)	Ref^d^.	0	Ref^d^.
UF-5000	BACT parameter^e^	87.3%	76.5%	53.9%	95.0%	0.819 (0.768-0.870)	7.32×10^-7^	37.4 (15.0-57.8)	6.04×10^-4^
	WBC parameter^f^	95.5%	54.0%	40.8%	97.3%	0.747 (0.705-0.790)	0.81	-1.9 (-17.5~13.7)	1.00
	BACT and WBC	84.1%	80.0%	57.0%	94.1%	0.821 (0.767-0.874)	1.32×10^-10^	39.9 (19.4-60.4)	1.36×10^-4^
	BACT or WBC	98.5%	51.0%	40.0%	99.0%	0.748 (0.710-0.785)	0.82	-3.4 (19.0~12.2)	0.66

PPV, positive predictive value; NPV, negative predictive value; AUC, area under the receiver operating curve; NRI, net reclassification index; CI, confidence interval; NIT, nitrite; BACT, bacteria count; WBC, white blood cells in urine; Ref., reference.

a Compare NIT and BACT.

b Compare dipstick WBC and UF-5000 WBC.

c Compare NIT and WBC and BACT and WBC.

d Compare NIT/WBC and BACT/BACT.

e Parameter (+) was set as BACT ≥196 units/μl.

f WBC parameter (+) was set as WBC≥14.7 cells/μl.

Among all enrolled patients, the concordance rate between UF-5000 and the MSU culture for the detection of bacteria was 59.0%. In patients without antibiotic treatment in 2 weeks prior to inclusion, the concordance rate was 65.4% (**Table 5** **)**. When predicting G+/G- bacteriuria, 38.3% of the results from UF-5000 were concordant with the MSU culture. Meanwhile, UF-5000 would have a higher concordance rate with the MSU culture (95.1%) when predicting a negative culture result. In patients without a prior use of antibiotics, the concordance rates between UF-5000 and culture for predicting G+/G- bacteriuria and a negative culture were 44.7% and 96.2%, respectively, which were higher than those of recently treated patients (**Table 5**). If excluding the mixed bacteriuria results, the overall concordance rate between UF-5000 and culture was higher in antibiotic-naïve patients (78.1%), symptomatic patients (73.3%), low-WBC patients (88.7%), and male patients (75.6%) (**Supplementary Tables 2–4**).

**Table 5:** Concordance rate of bacteria’s Gram pattern in different bacteria concentrations by UF-5000.

Group	Gram (+)/Gram(-)	Mixed	Undetectable	overall
**All patients (n=383)**				
Negative bacteriuria^a^	5.9%	0.0%	95.1%	76.9%
Positive bacteriuria^b^	38.3%	7.9%	100.0%	43.7%
Overall	33.9%	4.6%	95.1%	59.0%
Without mixed	–	–	–	70.5%
**Patients without having antibiotic treatments 2 weeks prior to inclusion (n=269)**				
Negative bacteriuria^a^	8.3%	0.0%	96.2%	88.8%
Positive bacteriuria^b^	44.7%	3.7%	100.0%	30.3%
Overall	39.8%	2.3%	96.2%	65.4%
Without mixed	–	–	–	78.1%
**Patients having antibiotic treatments 2 weeks prior to inclusion (n=104)**				
Negative bacteriuria^a^	0.0%	0.0%	92.0%	71.9%
Positive bacteriuria^b^	18.5%	20.0%	100.0%	18.9%
Overall	21.2%	14.3%	92.0%	52.5%
Without mixed	–	–	–	63.9%

MSU, midstream urine culture.

a Negative bacteriuria: bacteria count by UF-5000<196/μl.

b Positive bacteriuria: bacteria count by UF-5000≥196/μl.

The cutoff points of the BACT parameter (196/μl) were determined by the Youden index.

## Discussion

The performance of UF-5000 in UTI diagnosis from the emergency department is poorly examined or studied. In the present study, we found that UF-5000 urinalysis had a significantly better predictive value than another rapid test/analysis, the dipstick urine test, for predicting UTIs. In addition, it could provide bacterial discrimination for G+ or G- with acceptable concordance rates compared with the MSU culture, especially for predicting bacteria-negative samples.

The performance of UF-5000 is of significant clinical importance. First, a suspicious UTI patient from the emergency department may need immediate and effective intervention. Second, the MSU culture is time consuming, which usually costs days to perform, while UF-5000 may provide a quick reference for UTIs (including WBCs and the bacteriuria of G+/G-) within minutes. This may help to initiate a personalized targeted antibiotic treatment prior to the evidence obtained from the urine culture (days later). Third, the rapid result from UF-5000 may also potentially help to reduce the unnecessary antimicrobial treatment. In the current study, we were not able to evaluate whether UF-5000 urinalysis could reduce the overtreatment of antibiotics. However, the relatively high negative predictive value (NPV>94%), as well as the high concordance rate for predicting a negative MSU culture (>95%), could provide preliminary evidence for the further application of UF-5000. For instance, a delayed/non-antibiotic treatment may be provided in patients with negative results from UF-5000. Whether this potential application may bring benefits by reducing antibiotic overtreatment for UTIs will be further evaluated in our future studies.

Another important finding from this study should also be noted. Based on two previous studies conducted by the College of American Pathologists in 1998 (Valenstein and Meier, 1998) and 2008 (Bekeris et al., 2008), a urine specimen should be considered contaminated if the result of the culture indicates more than two isolates in quantities greater than or equal to 10,000 CFU/ml (Bekeris et al., 2008). One of the studies found that the contamination rates of urine specimens up to 41.7% (low-performance facilities), 15% (median performers), and 0.8% (high performers) correspond to the 10th, 50th, and 90th percentiles of facilities, respectively (Larocco et al., 2016). In addition, it is hard to redo the culture under this circumstance due to the use of antibiotics after initial MSU collection. Thus, in clinical practice, we need a rapid testing instrument used for filtering target patients and reducing unnecessary urine cultures. Other than the UF-5000’s proven capacity of screening out target patients by bacterial counts and WBC counts (Jolkkonen et al., 2010; Kim et al., 2018), we also evaluated the performance of the UF-5000 flagging system in discriminating Gram-negative or Gram-positive bacteria or mixed flora. In the present study, 44 out of 269 antibiotic-naïve patients (16.4%) and 69 out of 383 overall patients (18.0%) were reported with a mixture of microorganisms in urine by UF-5000. The concordant rate was increased obviously after removing those mixed samples that we defined as suspected contaminated samples. Although the sequential follow-up results of these patients were missing, multiple urine samples should be considered for these patients to obtain a more accurate result from the MSU culture. Therefore, UF-5000 urinalysis may also be used to identify and evaluate cleaning urine specimens quickly in detecting changes in bacterial species and bacterial burden and to increase the accuracy of urine culture microbiology.

There are several limitations of this study. First, the sample size was relatively small and a great part of the enrolled patients was not diagnosed as UTIs merely based on the urine culture results. Second, the gender distribution and urine sample types were unbalanced in the study. Affected by our microbiology lab reports, it was hard to tell if these catheter samples at <10^4^ CFU/ml were significant bacteriuria, which usually had a relatively lower threshold at 10^3^ CFU/ml. A future study with a larger sample size of UTIs and stricter criteria (e.g., more catheter samples with meticulous bacterial concentration classification) would be necessary to validate and enrich our findings. Third, the relatively low concordance rates were observed for predicting G+ and G- bacteriuria between UF-5000 and the MSU culture. This is another important issue that may also be due to the high rate of false negatives by MSU, especially in symptomatic women or patients who have already taken antibiotics (Chu and Lowder, 2018). Multiple urine sample collection and more comprehensive sequential follow-up should be considered in the design of future studies to better evaluate the function of UF-5000 as a complementary instrument to a regular MSU culture.

## Conclusion

UF-5000 urinalysis had a significantly better predictive value than the dipstick urine test for predicting UTIs. In addition, it could provide bacterial discrimination for G+ or G-, which might be helpful for initiating an antibiotic treatment in clinical practice.

## Data availability statement

The original contributions presented in the study are included in the article/**Supplementary Material**. Further inquiries can be directed to the corresponding authors.

## Ethics statement

This study was reviewed and approved by the institutional review board of the University of Hong Kong, and Hospital Authority Hong Kong West Cluster. The patients/participants provided their written informed consent to participate in this study.

## Author contributions

RN and H-LW conceived of and designed the study. RN, TC and XR processed and analyzed the data. XR, RN, TC, W-KM, WL, H-LW and TL helped with data collection and interpretations. H-LW, BH, CT, TL and AN made a critical revision of the manuscript for important intellectual content. RN, XR and TC wrote the manuscript. All authors contributed to the article and approved the submitted version.

## Acknowledgments

We would like to thank the participants in this study.

## Conflict of interest

Sysmex Corporation provided the reagent and labor cost (research assistant) for the study but did not participate in any other part of the study, including data analysis, result interpretation, and manuscript drafting. Blind data analysis and result interpretation were performed by XR from another institute (who did not receive any support from the company) with deidentified data.

The remaining authors declare that the research was conducted in the absence of any commercial or financial relationships that could be construed as a potential conflict of interest.

## Publisher’s note

All claims expressed in this article are solely those of the authors and do not necessarily represent those of their affiliated organizations, or those of the publisher, the editors and the reviewers. Any product that may be evaluated in this article, or claim that may be made by its manufacturer, is not guaranteed or endorsed by the publisher.
