# Supplementary material for: The diagnostic value of rapid urine test platform UF-5000 for suspected urinary tract infection at the emergency department
Source: Front Cell Infect Microbiol. 2022 Sep 27;12:936854. doi: 10.3389/fcimb.2022.936854 (PMC9551190; doi:10.3389/fcimb.2022.936854)
Supplement: Supplementary file 1 [file Table_1.docx]

**Supplementary table 1. Microorganisms identified in the 90 culture positive samples.**

| Microorganism identified | No. | % |
| --- | --- | --- |
| Escherichia coli | 46 | 51.11 |
| Enterococcus faecalis | 11 | 12.22 |
| Staphylococcus species | 6 | 6.67 |
| Streptococcus species | 6 | 6.67 |
| Klebsiella pneumonia | 6 | 6.67 |
| Pseudomonas aeruginosa | 4 | 4.44 |
| Proteus species | 3 | 3.33 |
| Nesseria species | 2 | 2.22 |
| Citrobacter species | 2 | 2.22 |
| Diphtheroids | 2 | 2.22 |
| Candida tropicalis | 1 | 1.11 |
| Morganella morganii | 1 | 1.11 |

**Supplementary table 2. Subgroup analysis of symptomatic and asymptomatic patients in concordance rate of bacteria’s gram pattern by UF5000.**

| Group | Gram (+)/Gram(-) | Mixed | Undetectable | overall |
| --- | --- | --- | --- | --- |
| **Symptomatic patients^a^ (n=147)** | | | | |
| Negative bacteriuria^b^ | 16.7% | 0.0% | 92.3% | 80.2% |
| Positive bacteriuria^c^ | 41.7% | 6.7% | 100.0% | 31.3% |
| Overall | 38.1% | 4.5% | 92.3% | 62.7% |
| Without mixed | - | - | - | 73.3% |
| **Asymptomatic patients (n=104)** | | | | |
| Negative bacteriuria^a^ | 0.0% | 0.0% | 95.3% | 69.5% |
| Positive bacteriuria^b^ | 38.2% | 16.7% | 100.0% | 35.0% |
| Overall | 35.1% | 5.3% | 95.3% | 55.6% |
| Without mixed | - | - | - | 67.5% |

Abbreviation: MSU, Midstream urine culture.

a. Symptomatic patient was defined as patients with symptoms such as dysuria, loin pain and fever.

b. Negative bacteriuria: bacteria count by UF5000<196/μL

c. Positive bacteriuria: bacteria count by UF5000≥196/μL

**Supplementary table 3. Subgroup analysis of different level of WBC parameter in concordance rate of bacteria’s gram pattern by UF5000.**

| Group | Gram (+)/Gram(-) | Mixed | Undetectable | overall |
| --- | --- | --- | --- | --- |
| **WBC parameter (+)^a^ (n=233)** | | | | |
| Negative bacteriuria^b^ | 9.1% | 0.0% | 89.7% | 58.2% |
| Positive bacteriuria^c^ | 42.7% | 12.5% | 100.0% | 34.3% |
| Overall | 39.5% | 8.2% | 89.7% | 43.8% |
| Without mixed | - | - | - | 56.4% |
| **WBC parameter (-) (n=150)** | | | | |
| Negative bacteriuria | 0.0% | 0.0% | 97.6% | 88.6% |
| Positive bacteriuria | 22.2% | 0.0% | 100.0% | 20.0% |
| Overall | 13.3% | 0.0% | 97.6% | 84.0% |
| Without mixed | - | - | - | 88.7% |

Abbreviation: MSU, Midstream urine culture.

a. WBC parameter (+) was set as WBC by UF-5000≥14.7 cells/μL.

b. Negative bacteriuria: bacteria count by UF5000<196/μL.

c. Positive bacteriuria: bacteria count by UF5000≥196/μL.

**Supplementary table 4. Subgroup analysis of sex in concordance rate of bacteria’s gram pattern by UF5000.**

| Group | Gram (+)/Gram(-) | Mixed | Undetectable | overall |
| --- | --- | --- | --- | --- |
| **Male (n=264)** | | | | |
| Negative bacteriuria^a^ | 0.0% | 0.0% | 97.2% | 80.6% |
| Positive bacteriuria^b^ | 32.8% | 8.3% | 100.0% | 25.9% |
| Overall | 29.4% | 4.3% | 97.2% | 62.7% |
| Without mixed | - | - | - | 75.6% |
| **Female (n=109)** | | | | |
| Negative bacteriuria | 11.1% | 0.0% | 91.1% | 66.7% |
| Positive bacteriuria | 47.6% | 7.1% | 100.0% | 37.5% |
| Overall | 41.1% | 5.3% | 91.1% | 51.0% |
| Without mixed | - | - | - | 61.2% |

Abbreviation: MSU, Midstream urine culture.

a. Negative bacteriuria: bacteria count by UF5000<196/μL.

b. Positive bacteriuria: bacteria count by UF5000≥196/μL.
